# Diversity of Na^+^ allocation in salt-tolerant species of the genus *Vigna*

**DOI:** 10.1270/jsbbs.22012

**Published:** 2022-08-30

**Authors:** Yusaku Noda, Ryohei Sugita, Atsushi Hirose, Naoki Kawachi, Keitaro Tanoi, Jun Furukawa, Ken Naito

**Affiliations:** 1 Takasaki Advanced Radiation Research Institute, National Institutes for Quantum Science and Technology (QST), 1233 Watanuki-machi, Takasaki, Gunma 370-1292, Japan; 2 Research Center of Genetic Resources, National Agriculture and Food Research Organization, 2-1-2 Kannondai, Tsukuba, Ibaraki 305-8602, Japan; 3 Radioisotope Research Center, Nagoya University, Furo-cho, Chikusa, Nagoya, Aichi 464-8602, Japan; 4 Hoshi University, 2-4-41 Ebara, Shinagawa, Tokyo 142-8501, Japan; 5 Graduate School of Agricultural and Life Sciences, The University of Tokyo, 1-1-1 Yayoi, Bunkyo, Tokyo 113-8657, Japan; 6 Faculty of Life and Environmental Sciences, University of Tsukuba, 1-1-1 Tennodai, Tsukuba, Ibaraki 305-8572, Japan

**Keywords:** genus *Vigna*, genetic resources, wild crop relatives, autoradiography, salt tolerance

## Abstract

Wild species in the genus *Vigna* are a great resource of tolerance to various stresses including salinity. We have previously screened the genetic resources of the genus *Vigna* and identified several accessions that have independently evolved salt tolerance. However, many aspects of such tolerance have remained unknown. Thus, we used autoradiography with radioactive sodium (^22^Na^+^) and Inductively Coupled Plasma Mass Spectrometry (ICP-MS) to visualize and compare Na^+^ allocation in *Vigna angularis* (Willd.) Ohwi & H.Ohashi (azuki bean), *Vigna nakashimae* (Ohwi) Ohwi & H.Ohashi, *Vigna riukiuensis* (Ohwi) Ohwi & H.Ohashi, *Vigna luteola* (Jacq.) Benth. and *Vigna marina* (Burm.) Merr.. The results indicated: 1) Tolerant accessions suppress Na^+^ accumulation compared to azuki bean. 2) *V. nakashimae* and *V. marina* does so by accumulating higher amount of K^+^, whereas *V. riukiuensis* and *V. luteola* does so by other mechanisms. 3) *V. luteola* avoids salt-shedding by allocating excess Na^+^ to newly expanded leaves. As the mechanisms of the tolerant species were different, they could be piled up in a single crop *via* classical breeding or by genetic engineering or genome editing.

## Introduction

Salt tolerance is an important issue given more and more arable lands are degraded by soil salinity. In addition, ground water has been rapidly depleted especially in areas where people run agriculture with intensive irrigation (Wada *et al.* 2010). As such, there is a growing demand for salt-tolerant crops that can be grown in saline soil or with saline water (Panta *et al.* 2014).

However, improving salt tolerance has been a challenging issue for plant scientists. One of the reasons is low diversity of salt tolerance in domesticated species, which is especially true to legume crops. Extensive screening for salt tolerance has been performed, but such efforts have identified few accessions that are potentially useful (Akita and Cabuslay 1990, Farooq and Azam 2006, Katerji *et al.* 2006).

This is why wild genetic resources have recently been recognized as important source of stress tolerance (McCouch *et al.* 2013). Among plant taxa, we have been focusing the genus *Vigna* because of its great diversity and adaptability to harsh environments (Tomooka *et al.* 2014, van Zonneveld *et al.* 2020). Moreover, many of the wild species are relatives of important crops, such as cowpea (*Vigna unguiculata* (L.) Walp.), mung bean (*Vigna radiata* (L.) R. Wilczek), and azuki bean (*Vigna angularis* (Willd.) Ohwi and H.Ohashi). As such, once a stress-tolerant accession is identified, it can be directly used for cross-breeding. In addition, if the genes responsible for the stress tolerance are identified, broader application will be possible through genetic engineering or genome editing.

Thus, we have previously screened the wild species of the genus *Vigna* and identified several accessions that are highly tolerant to salt stress (Iseki *et al.* 2016, Yoshida *et al.* 2016, 2020). In these screenings, we evaluated salt tolerance by wilting score (visually) (Yoshida *et al.* 2016, 2020) and chlorophyll fluorescence (Iseki *et al.* 2016). In addition, some of these species grew even better in a salinized field by Tsunami than in a de-salinized field in Fukushima, where modern soybean cultivars did not grow at all (Yoshida *et al.* 2016).

However, mechanisms of salt tolerance in these salt-tolerant species are almost unknown. Given phylogenetic relationship indicates the tolerant species have independently evolved salt tolerance (Iseki *et al.* 2016), it is important to elucidate whether they have acquired mechanisms that are similar to or different from each other. If the former is the case, we do not need to analyze all the tolerant accessions but one representative species. If the latter is the case, there will be a possibility of introducing multiple mechanisms of salt tolerance to develop a super-tolerant crop. This can be achieved even by cross-breeding if the tolerant species are genetically close enough to the target crop. Isolation of responsible genes would make super-tolerance more achievable, although the target crop needs transformation technique to be established.

Thus, to elucidate differences in the mechanisms of salt tolerance among the tolerant species, we surveyed Na^+^ accumulation and allocation in the plants using ^22^Na^+^, a radio-isotope (RI) of Na^+^, and autoradiographic techniques. We tested five species, of which four are salt-tolerant and one is salt-sensitive. The tolerant species are *Vigna nakashimae* (Ohwi) Ohwi & H.Ohashi, *Vigna riukiuensis* (Ohwi) Ohwi & H.Ohashi, *Vigna luteola* (Jacq.) Benth. and *Vigna marina* (Burm.) Merr. that are tolerant to 100, 150, 300 and 400 mM NaCl, respectively (Yoshida *et al.* 2016, 2020). The sensitive species is *Vigna angularis* (Willd.) Ohwi and H.Ohashi (azuki bean), which is severely damaged even in 50 mM NaCl (Iseki *et al.* 2016, Yoshida *et al.* 2016). The results revealed that the salt-tolerant species have different patterns of Na^+^ allocation and accumulation, indicating the mechanisms of salt tolerance are different from each other.

## Materials and Methods

### Plant material and growth conditions

Table 1 summarizes the species names, the accession numbers and the periods of preculture of all the materials tested in this study. All the seeds were provided from the NARO Genebank in Tsukuba, Japan (https://www.gene.affrc.go.jp/index_en.php). Seeds were germinated on Seramis clay (Westland Deutschland GmbH, Mogendorf, Germany) for 1 week and then transferred to hydroponic solution in a growth chamber (Light: 28°C for 14 h and Dark: 25°C for 10 h. Light intensity 500 μM^–1^ s^–1^ m^–2^). Hydroponic solution contained diluted nutrient solution of a 1:1 ratio of OAT House No.1 (1.5 g L^–1^): OAT House No.2 (1 g L^–1^) (Otsuka Chemical Co., Japan), which contained 18.6 mEq L^–1^ N, 5.1 mEq L^–1^ P, 8.6 mEq L^–1^ K, 8.2 mEq L^–1^ Ca and 3.0 mEq L^–1^ Mg.

### Tracer experiment of ^22^Na^+^

Supplemental Fig. 1 summarizes procedure of autoradiography, which we performed in Center for Research in Isotopes and Environmental Dynamics in University of Tsukuba, Japan. Pre-cultured plant was transplanted to new hydroponic solution containing 100 kBq ^22^Na^+^ (PerkinElmer, USA) with non-radioactive 100 mM ^23^NaCl. After adding the radio-isotope, plants were incubated again in a long-day condition (Light: 28°C for 14 h and Dark: 25°C for 10 h. Light intensity 200 μmol s^–1^ m^–2^) for 3 or 6 days. After incubation, we carefully washed the roots and then enclosed the whole plant body into plastic bags and exposed it to a Storage Phosphor Screen (BAS-IP-MS-2025E, GE Healthcare, UK) in Amersham exposure cassettes (GE Healthcare, UK) for 24 h. We then scanned the exposed screen with a laser imaging scanner Typhoon FLA-9500 (GE Healthcare, UK). To arrange radioactive intensity equally at each image, photo-stimulated luminescence and contrast were equalized by Multi Gauge *ver.* 3.0 (Fujifilm, Japan). Then, we separated root, shoot and leaves, measured the fresh weight, and dried the samples at 50°C for 3 days. The dried samples were measured for the ^22^Na^+^ radioactivity with the gamma counters AccuFLEX γ7001 (Hitachi Aloka Medical, Japan) as well as dry weight. All the experiments were independently done with three or four biological replicates. All the evaluated values were tested with Tukey’s honestly significant difference (HSD) test by multcomp package for R (Hothorn *et al.* 2008). Differences were significant when p < 0.05.

### ICP-MS

We germinated the seeds on Seramis clay, cultivated for 1 week and then transferred 4 plants of each species to hydroponic solution (as described above) in a growth chamber (Light: 28°C for 14 h and Dark: 24°C for10 h). When the 3^rd^ leaves had fully expanded, we transferred the plants to hydroponic culture with 100 mM NaCl for 2 days. After incubation, we separately collected the 1^st^ and the 2^nd^ leaves and dried at 50°C for 3 days. The leaves were digested with 200 μL 69% HNO_3_ at 90°C for 0.5 h. The digestate was diluted 1-in-140 with Milli-Q water and inductively coupled plasma-mass spectrometry (ICP-MS, NexION 350S, PerkinElmer, Waltham, MA, USA) determined the contents of Na^+^ and K^+^, respectively. The Tukey HSD of statistical analysis was used to compare differences in the measured variables of leaf Na^+^ and K^+^ concentration, respectively. Differences were significant when p < 0.05.

## Results

### Na^+^ allocation in salt-sensitive and salt-tolerant species

**Fig. 1.**
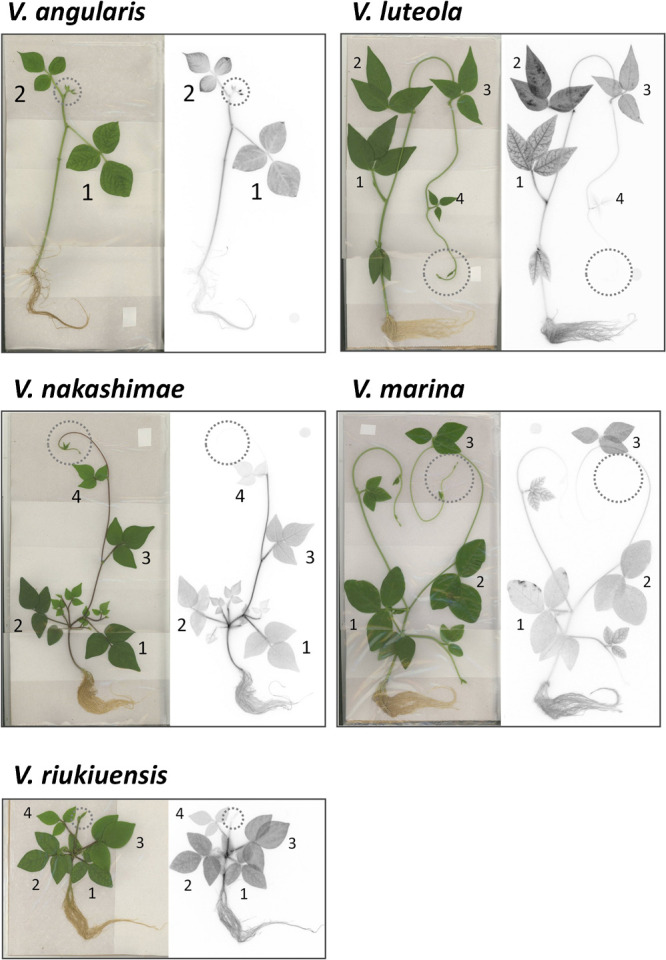
Autoradiography of ^22^Na-treated plants. Photos were taken after 72 h treatment with 100 mM NaCl containing ^22^Na^+^. Numbers indicate the 1^st^, 2^nd^, 3^rd^ and 4^th^ leaves, respectively. Dotted circles indicate locations of the shoot apices.

**Fig. 2.**
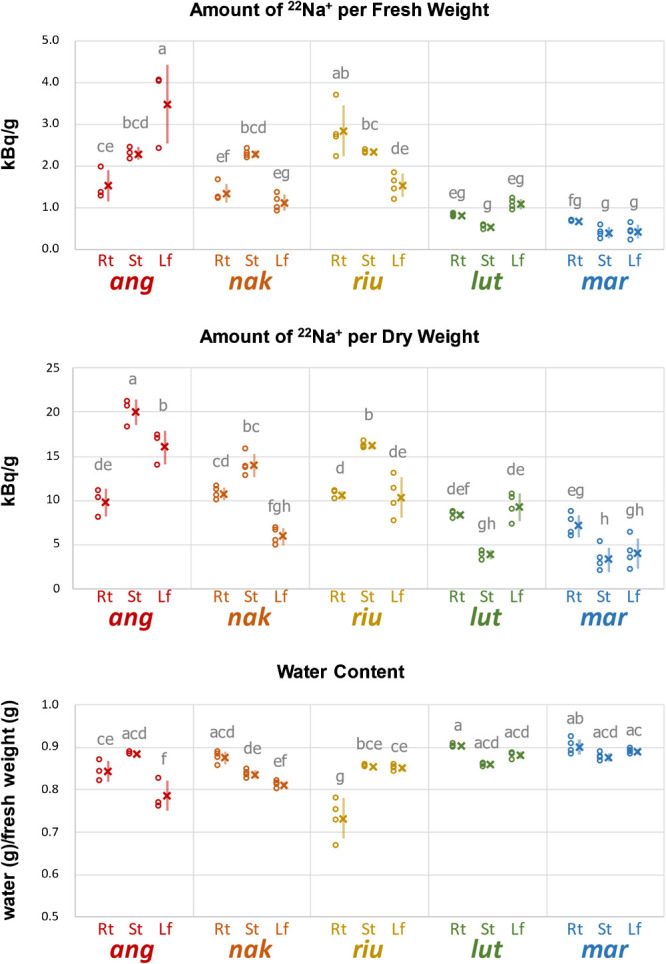
Amount of ^22^Na per biomass and water content in the roots, stems and leaves. Open circles, X and error bars indicate values of each replicate, means and standard deviations, respectively. Rt, St and Lf indicate root, stem and leaf, respectively. *ang*, *nak*, *riu*, *lut* and *mar* indicate *V. angularis*, *V. nakashimae*, *V. riukiuensis*, *V. luteola* and *V. marina*, respectively. Means not sharing the same alphabet are significantly different (Tukey HSD p < 0.05).

We performed ^22^Na^+^ tracer experiment by treating the plants with 100 mM NaCl including ^22^Na^+^ for 3 days in hydroponic culture. The following autoradiography revealed similarities and differences of ^22^Na^+^ localization in the tolerant accessions (Fig. 1). Note that autoradiography is suitable for visualizing differences in ^22^Na^+^ allocation within an image, but is not for visualizing differences between images. For example, although the image of *V. luteola* in Fig. 1 is darker than that of *V. angularis*, it does not mean *V. luteola* accumulated more ^22^Na^+^ than *V. angularis* (see Fig. 2). After taking autoradiography, we evaluated ^22^Na^+^ allocation per biomass (gram fresh weight and gram dry weight), based on the count data of irradiation (Fig. 2).

Overall, the count data indicated ^22^Na^+^ allocation was higher in azuki bean, *V. nakashimae* and *V. riukiuensis*, and lower in *V. luteola* and *V. marina* (Fig. 2). The trend was particular in the stem, where the former three accumulated more ^22^Na^+^ in the stem than in the root, whereas the latter two accumulated more in the root than in the stem (Fig. 2).

In azuki bean, which was the only salt-sensitive species in this study, ^22^Na^+^ allocation to the root was lower than those to the stem or to the leaf (Fig. 1). As shown in Fig. 2, the amount of ^22^Na^+^ per biomass was by 50–100% higher in the leaf or in the stem than in the root. In addition, ^22^Na^+^ was highly accumulated in the tips of the 2^nd^ leaf and in the shoot apex, whereas it was not so in the veins of the 1^st^ leaf (Fig. 1).

In *V. nakashimae*, ^22^Na^+^ was less allocated to the leaf compared to the stem and the root (Fig. 1). In contrast with azuki bean, ^22^Na^+^ was not allocated to the shoot apex (Fig. 1). The lower ^22^Na^+^ allocation to the leaf was confirmed by evaluating the amount of ^22^Na^+^ per biomass of both fresh weight and dry weight (Fig. 2).

The autoradiography of *V. riukiuensis* showed a similar pattern of ^22^Na^+^ allocation with that of *V. nakashimae* (Fig. 1). However, in the context of per fresh weight, ^22^Na^+^ allocation was higher in the root than in the leaf (Fig. 2). In the context of per dry weight, allocation to the stem was significantly higher than to the leaf or the root (Fig. 2).

*V. luteola* showed a unique pattern of ^22^Na^+^ allocation, where the 2^nd^ leaf accumulated more ^22^Na^+^ than the 1^st^ or other leaves (Fig. 1). Interestingly, more ^22^Na^+^ was allocated in the mesophylls of the 2^nd^ leaf, whereas it was more allocated in the veins of the 1^st^ leaf (Fig. 1). In addition, ^22^Na^+^ was not allocated to the shoot apex (Fig. 1). In the context of per fresh weight, ^22^Na^+^ allocation was significantly the most to the leaf and the least to the stem (Fig. 2). In the context of dry weight, allocation to the leaf or the root was significantly higher than to the stem (Fig. 2).

*V. marina* mainly allocated ^22^Na^+^ to the root and not to the shoot apex (Fig. 1). In both contexts of per fresh and dry weight, ^22^Na^+^ was significantly higher in the root than in the stem or the leaf (Fig. 2).

### Water content

Because succulence is one of the well-known responses to salt stress to dilute the negative effect of Na^+^ in fresh tissues (Aziz and Khan 2001), we calculated water content of each sample based on the subtraction of the dry weight from the fresh weight. Overall, water content negatively correlated with Na^+^ allocation per fresh weight. The lowest water content was observed in the leaf of azuki bean and the root of *V. riukiuensis*, both of which showed the highest Na^+^ allocation per fresh weight (Fig. 2). On the other hand, higher water content was observed in the roots and the leaves of *V. luteola* and *V. marina*, which showed lower Na^+^ allocation per fresh weight than others (Fig. 2).

### Na^+^/K^+^ Ratio in the old and new leaves

**Fig. 3.**
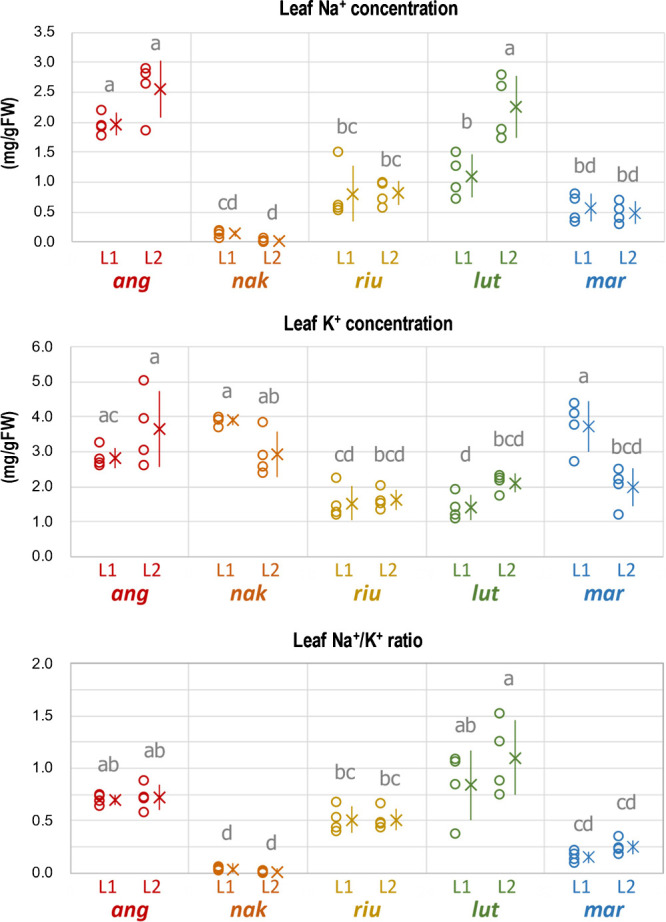
Concentrations of Na^+^ and K^+^ and Na^+^/K^+^ ratio in the 1^st^ and the 2^nd^ leaves of the plants. The plants were treated with 100 mM NaCl for 2 days, and then sampled for ICP-MS. Open circles, X and error bars indicate values of each replicate, means and standard deviations, respectively. L1 and L2 indicate the 1^st^ and 2^nd^ leaves, respectively. *ang*, *nak*, *riu*, *lut* and *mar* indicate *V. angularis*, *V. nakashimae*, *V. riukiuensis*, *V. luteola* and *V. marina*, respectively. Means not sharing the same alphabet are significantly different (Tukey HSD p < 0.05).

To elucidate often-mentioned Na^+^/K^+^ homeostasis, we measured the concentration (mg per gram fresh weight) of Na^+^ and K^+^ in the 1^st^ and the 2^nd^ leaves after cultivating the plants in 100 mM NaCl for 48h using ICP-MS. The results reproduced the overall trend of Na^+^ allocation observed in the tracer experiment (Figs. 1, 2), where the Na^+^ concentration in azuki bean was again significantly higher compared to those in the tolerant species (Fig. 3). The results also reproduced a characteristic pattern of Na^+^ allocation of *V. luteola*, which allocated more Na^+^ to the 2^nd^ leaf than to the 1^st^ although *V. nakashimae* showed even lower Na^+^ concentration compared to the tracer experiment (Figs. 2, 3). Except *V. nakashimae* and the 2^nd^ leaf of *V. luteola*, Na^+^ concentration was not significantly different between the leaves of tolerant species.

There were also some variations in K^+^ concentration (Fig. 3). Azuki bean, *V. nakashimae* and *V. marina* showed higher K^+^ concentration compared to *V. riukiuensis* and *V. luteola* (Fig. 3).

The calculated Na^+^/K^+^ ratio showed that only *V. nakashimae* and *V. marina* maintained low Na^+^/K^+^ ratio, whereas azuki bean, *V. riukiuensis* and *V. luteola* did not (Fig. 3).

### ^22^Na^+^ allocation in longer duration of salt stress

**Fig. 4.**
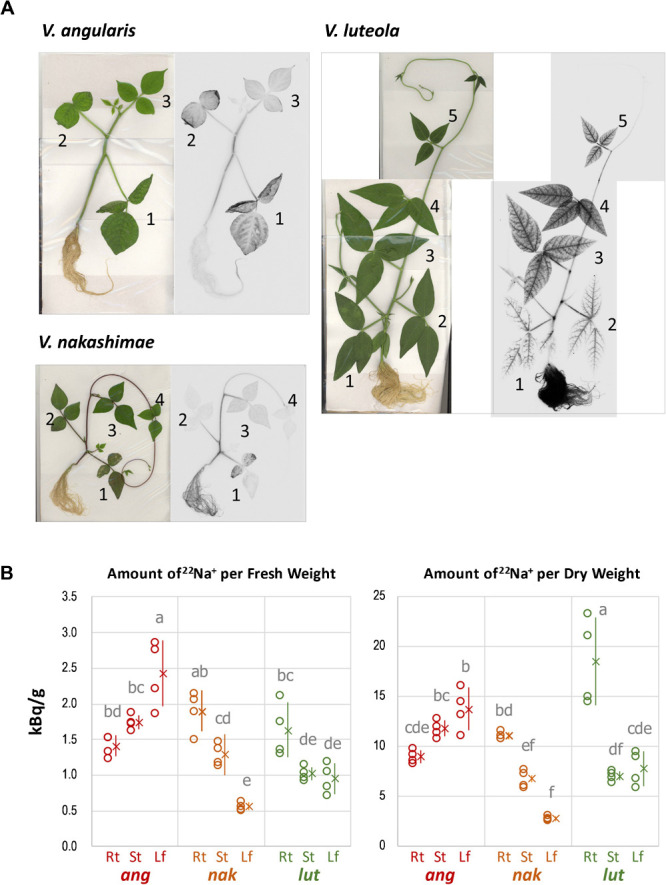
Autoradiography and irradiation count of ^22^Na-treated plants with longer duration time. A. Autoradiography. Photos were taken after 144 h treatment with 100 mM NaCl containing ^22^Na. Numbers indicate the 1^st^, 2^nd^, 3^rd^, 4^th^ and 5^th^ leaves, respectively. B. Amount of ^22^Na per biomass. Open circles, X and error bars indicate values of each replicate, means and standard deviations, respectively. Rt, St and Lf indicate root, stem and leaf, respectively. *ang*, *nak*, and *lut* indicate *V. angularis*, *V. nakashimae*, and *V. luteola*, respectively. Means not sharing the same alphabet are significantly different (Tukey HSD p < 0.05).

We also tested three species (azuki bean, *V. nakashimae* and *V. luteola*) with longer period (6 days) of ^22^Na^+^ feeding. As a result of longer duration of salt stress, azuki bean exhibited salt damage in the 1^st^ and the 2^nd^ leaves, where excess amount of ^22^Na^+^ was accumulated at the edge of the leaves (Fig. 4A). *V. nakashimae* also exhibited salt damage in the 1^st^ leaf, which accumulated more ^22^Na^+^ than other leaves (Fig. 4A). In contrast, *V. luteola* allocated less ^22^Na^+^ to the 1^st^ and 2^nd^ leaf, while it accumulated more ^22^Na^+^ in the 3^rd^ and 4^th^ leaves (Fig. 4A). In addition, *V. luteola* allocated a lot more ^22^Na^+^ to the root than to the leaf or the stem (Fig. 4A).

In addition, the longer duration time of salt stress induced a different pattern of Na^+^ allocation in the tolerant species (Fig. 4B). While azuki bean showed more Na^+^ allocation to the leaf even in the context of kBq/g dry weight, *V. nakashimae* and *V. luteola* clearly allocated more Na^+^ to the root, which contrasted from the pattern in Fig. 2 (Fig. 4B).

## Discussion

In this study, we demonstrated that the genus *Vigna* has developed diverse mechanisms of salt tolerance. Whereas azuki bean, which is salt-sensitive, cannot prevent Na^+^ allocation to the leaves and shoot apex, the tolerant species basically keep the shoot apex and leaves away from Na^+^ (Figs. 1, 2). However, our results indicated there are various options to achieve low Na^+^ allocation to the leaves.

The biggest difference among the tolerant species is in that *V. nakashimae* and *V. riukiuensis* use the stems to evacuate Na^+^, whereas *V. luteola* and *V. marina* use the roots to do so. In addition, *V. luteola* and *V. marina* have ability to suppress Na^+^ accumulation, as they accumulate significantly lower amount of Na^+^ in all the organs than the others (Figs. 2, 3). These results indicate Na^+^ evacuation by root may reduce total amount of Na^+^ loaded into xylem flow. However, as the plants get acclimated by longer duration of salt stress, Na^+^ evacuation in the root increases also in *V. nakashimae* (Fig. 4). But the ability of Na^+^ evacuation in the root is still lower compared to *V. luteola*, where Na^+^ allocation became even higher by longer duration of salt stress (Fig. 4).

Another difference among the tolerant species is in that the tolerant species prevent leaf Na^+^ allocation by using K^+^ as a competing cation or not. Although *V. nakashimae* and *V. riukiuensis* have significantly lower Na^+^ concentration in the leaf than azuki bean (Figs. 1–3), *V. nakashimae* does so by allocating more K^+^, whereas *V. riukiuensis* does not. Since K^+^ is monovalent cation as Na^+^ is, increasing K^+^ concentration in the cytosol prevents Na^+^ uptake (Zhu 2003). This is a mechanism called Na^+^/K^+^ homeostasis, which *V. nakashimae* might rely on. On the other hand, *V. riukiuensis* suppresses Na^+^ allocation to the leaf without maintaining low Na^+^/K^+^ ratio (Figs. 1, 2, 4). As such, this species might have other unknown mechanisms. *V. luteola* should also have unknown mechanisms to suppress Na^+^ allocation to the 1^st^ leaf, as its Na^+^/K^+^ ratio was even higher, though not significant, than *V. riukiuensis* (Fig. 3). Thus, *V. nakashimae* and *V. marina* have a good system of Na^+^/K^+^ homeostasis but others do not.

In addition, our data of water content (Fig. 2) indicated succulence could be one of the possible mechanisms other than Na^+^/K^+^ homeostasis. By increasing water content in the leaves, *V. riukiuensis*, *V. luteola* and *V. marina* dilute Na^+^ concentration and may buffer disruptive effect of Na^+^ on enzymatic activities (Aziz and Khan 2001). Given succulence requires maintenance of osmotic pressure, K^+^ might play some roles in maintaining the high water content in *V. marina* (Figs. 2, 3). On the other hand, *V. riukiuensis* and *V. luteola* may need other compatible solutes to do so.

We also found difference in the way dealing with excessively absorbed Na^+^. As the duration time of salt stress becomes longer, *V. nakashimae* mainly allocates Na^+^ to the 1^st^ leaf (Fig. 3), which will be shed to discard excess salt when saturated. This is so-called salt shedding, which is often observed in mangrove plants (Aziz and Khan 2001). In contrast, *V. luteola* allocates Na^+^ to the upper leaves and not to the 1st leaf. Although *V. luteola* allocates Na^+^ mainly to the 2^nd^ leaf in the 3-leaf stage (Figs. 1, 3), it does so to the 3^rd^ and 4^th^ leaves in the 5-leaf stage (Fig. 4). This indicates, as new leaves grow and expand, *V. luteola* changes from the older leaf to the newer one for loading excess Na^+^, having each leaf load Na^+^ for a temporary period of time. Thus, before the leaf is saturated with Na^+^, the next leaf grows and takes over the role of Na^+^ loading. The advantage of such “taking turns” system might be in that *V. luteola* does not have to sacrifice any leaves to discard excess Na^+^ unless salt stress is too severe to grow.

Here we summarize the mechanisms of Na^+^ allocation of the salt-tolerant species in the genus *Vigna*.

1. *V. marina*, the most salt-tolerant species in the genus, evacuates Na^+^ by the root and suppresses Na^+^ allocation to the stem and the leaf. It is also able to maintain high water content and low Na^+^/K^+^ ratio in the leaf, which may further contribute to lower Na^+^ allocation to the leaf.

2. *V. luteola* also evacuates Na^+^ by the root. Instead of relying on Na^+^/K^+^ homeostasis, it avoids salt shedding by changing leaves for Na^+^ loading. It also has an ability to maintain higher water content.

3. *V. riukiuensis* uses the stem to evacuate Na^+^ and thus accumulates relatively higher amount of Na^+^ compared to *V. marina* or *V. luteola*. It also suppresses Na^+^ allocation to the leaf and shoot apex by unknown mechanisms other than Na^+^/K^+^ homeostasis.

4. *V. nakashimae* also uses the stem to evacuate Na^+^ and suppresses Na^+^ allocation to the leaf and shoot apex by Na^+^/K^+^ homeostasis. However, the excess amount of Na^+^ will be allocated to the oldest leaf and discarded by salt shedding.

As described above, even within the genus *Vigna*, the wild species have acquired various mechanisms of salt tolerance. Given *V. nakashimae* and *V. riukiuensis* are crossable with azuki bean and rice bean (*Vigna umbellata* (Thunb.) Ohwi & H.Ohashi), it will be possible to introduce the mechanisms of both species into these grain legumes. We are also currently in a process of isolating genes of salt tolerance from *V. marina* and *V. luteola*, which will facilitate development of super-tolerant crop against salt stress. To this end, however, further analyses including genetics (Chankaew *et al.* 2014), genomics (Naito *et al.* 2022 (https://doi.org/10.1101/2022.03.28.486085), Sakai *et al.* 2016) and transcriptomics will be necessary.

## Acknowledgments

This study was financially supported by JSPS KAKENHI Grant Number 18H02182, JST PRESTO Grant Number 11103610, Moonshot R&D Program for Agriculture, Forestry and Fisheries by Cabinet Office, Government of Japan (20350204), Environmental Radioactivity Research Network Center (Y-19-05) and Interdisciplinary Project on Environmental Transfer of Radionuclides (No. Y-1).

## Author Contribution Statement

YN, KT, NK, JF and KN planned the research.

YN, RS and KT performed experiments.

YN, RS, KT and KN analyzed data.

AH and JF tested the results.

YN and KN wrote the paper.

## Supplementary Material

Supplemental Figure

## Figures and Tables

**Table 1.** Plant materials and pre-culture period

Species	Accession number	Salt tolerance	Pre-culture period (days) without NaCl
*V. angularis*	JP37752	Sensitive*^†^	10
*V. nakashimae*	JP107879	Tolerant*^†^	14
*V. riukiuensis*	JP108810	Tolerant*^†^	17
*V. luteola*	JP233389	Tolerant^†‡^	14
*V. marina*	JP235813	Tolerant^†‡^	17

* Evaluated by visual scoring (Yoshida *et al.* 2016). ^†^ Evaluated by chlorophyll fluorescence (Iseki *et al.* 2016). ^‡^ Evaluated by visual scoring (Yoshida *et al.* 2020).
