# Supplementary figures and images for: Diversity of Na+ allocation in salt-tolerant species of the genus Vigna
Source: Breed Sci. 2022 Aug 30;72(4):326–31. doi: 10.1270/jsbbs.22012 (PMC9868329; doi:10.1270/jsbbs.22012)

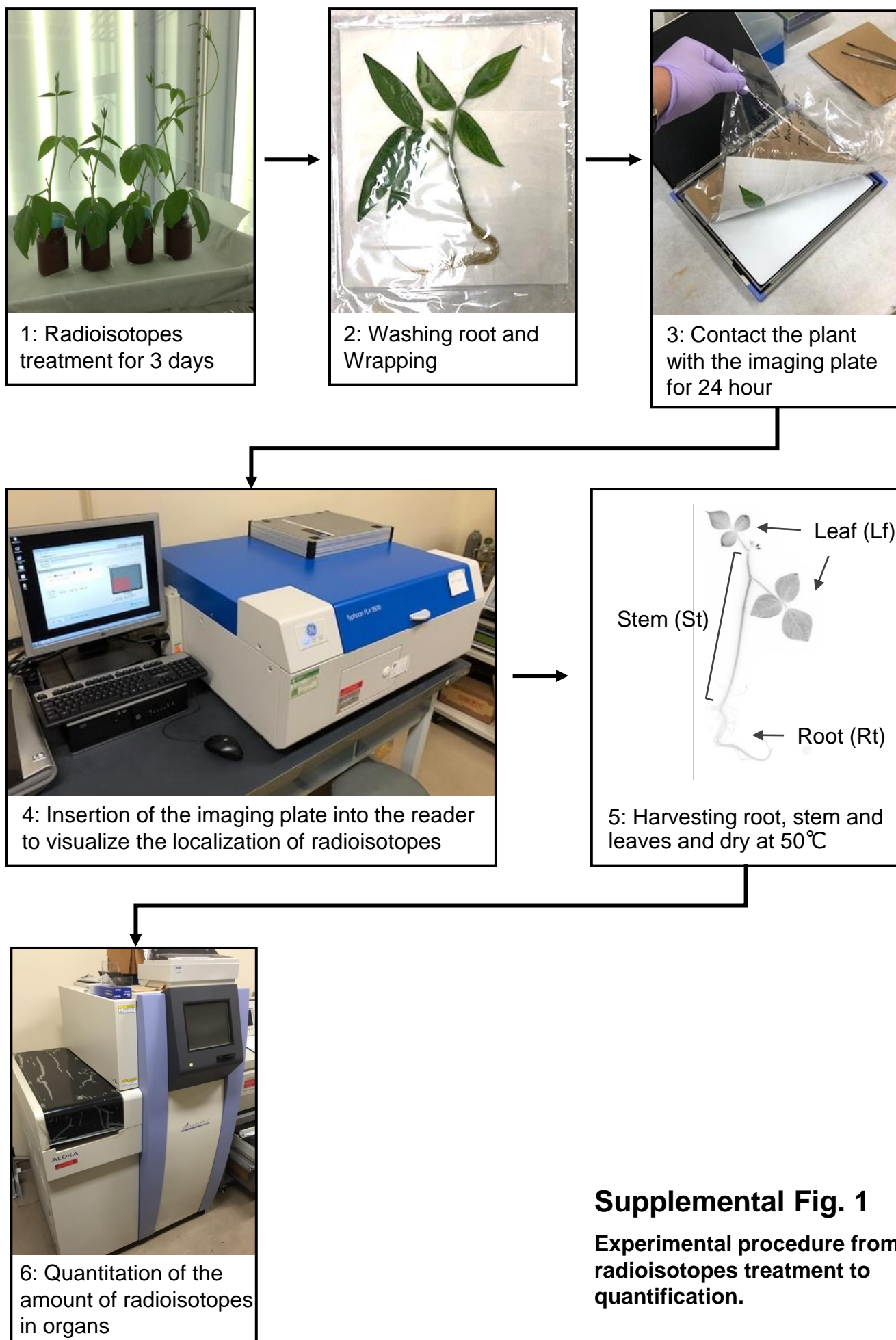

## Supplemental Fig. 1

Experimental procedure from radioisotopes treatment to quantification.

Supplement: Supplementary file 1 — Supplemental Figure [file 72_326_s1.pdf]
